# Acute changes in morphology and renal vascular relaxation function after renal denervation using temperature-controlled radiofrequency catheter

**DOI:** 10.1186/s12872-019-1053-z

**Published:** 2019-03-22

**Authors:** Enyong Su, Linwei Zhao, Chuanyu Gao, Wen Zhao, Xianpei Wang, Datun Qi, Lijie Zhu, Xiaohang Yang, Binbin Zhu, Yahui Liu

**Affiliations:** 1 Department of Cardiology, Zhengzhou University People’s Hospital, No.7 Weiwu road, Jinshui District, Zhengzhou, 450003 China; 2 Zhengzhou University School of Pharmaceutical Sciences, Zhengzhou, 450003 Henan China

**Keywords:** Histology, Radiofrequency ablation, Renal denervation, Endothelial dysfunction, Renal nerves, Renal arteries, Hypertension

## Abstract

**Background:**

Resistant hypertension and renal sympathetic hyperactivity are closely linked, and catheter-based renal denervation (RDN) is regarded as a new treatment strategy. However, the acute changes in vascular morphology and relaxation function have yet to be evaluated, and these may be important for the efficacy and safety of the procedure. In this study, we explored these questions by conventional temperature-controlled cardiac radiofrequency catheter-based RDN in a pig model.

**Methods:**

Six mini-pigs were randomly divided into the renal denervation (RDN) group (*n* = 3) and the Sham-RDN group (*n* = 3). Animals in the RDN group underwent unilateral radiofrequency ablation, and those in the Sham-RDN group underwent the same procedure except for the ablation. The pigs were examined by angiography pre- and post-RDN and were euthanized immediately thereafter. Renal arteries were processed for histological and molecular biology analyses as well as for in vitro vascular tension testing.

**Results:**

Compared with the Sham-RDN group, the RDN caused vascular intima and media injury, renal nerve vacuolization, mild collagen fiber hyperplasia and elastic fiber cleavage (all *p* < 0.05). The RDN group also significantly exhibited nitric oxide synthase pathway inhibition and decreased endothelium-independent vascular relaxation function Compared to the Sham-RDN group (all *p* < 0.05).

**Conclusions:**

In this porcine model, renal artery denervation led to vascular wall injury and endothelial dysfunction in the acute phase, which negatively affected vascular relaxation function. Thus, this process may be detrimental to the prognosis and progress of hypertension patients.

**Electronic supplementary material:**

The online version of this article (10.1186/s12872-019-1053-z) contains supplementary material, which is available to authorized users.

## Background

It is well known that hypertension seriously damages human health. Hypertension significantly increases the risk of cardiovascular and cerebrovascular diseases, such as coronary heart disease, heart failure, stroke, and chronic renal disease [1]. Despite substantial efforts to improve hypertension, such as a healthy lifestyle, regular exercise, diet regulation and a variety of available antihypertensive drugs targeted at different pathophysiological mechanisms, there is a special type of refractory hypertension called resistant hypertension, which is defined as occurring when a patient takes three or more antihypertensive drugs, including a diuretic, at optimal tolerated doses but fails to achieve blood pressure control [2–4]. Patients with resistant hypertension have been reported to account for 5–30% of all hypertensive patients [5], indicating the need for a new therapeutic approach for hypertension.

Overactivity of the sympathetic nervous system is involved in the onset of resistant hypertension [6]. Efferent renal sympathetic nerves overactivity promotes the production of norepinephrine, causes renal vasoconstriction and reduces renal blood flow, which gives rise to activation of the renin-angiotensin-aldosterone system and causes water retention, sodium reabsorption; Excessive activation of afferent fibers can activate the systemic sympathetic system through the central sympathetic nervous system, causing changes in the structure and function of target organs such as kidneys, heart and blood vessels and exacerbating the degree of hypertension [7, 8]. Therefore, innovative treatment of the sympathetic nervous system may be effective for treating resistant hypertension. In recent years, radiofrequency catheter-based renal denervation (RDN) has been successfully applied for treating resistant hypertension [9–11]. Additionally, early nonrandomized clinical trials have shown a relation between RDN and decreased blood pressure [12, 13]. However, failure to meet the efficacy end point was observed in the Simplicity HTN-3 study, challenging the results of the Simplicity HTN-1 and 2 studies [14, 15].

Moreover, the ablation energy may damage the vascular wall, which plays an important role in vascular function. Although there are a small number of preclinical studies on the effects of catheter-based RDN [16–19], the acute alterations in vascular relaxation function after RDN have not been explored. In addition, the changes assessed immediately after RDN of the renal arterial wall and renal nerves are inadequate, which may be essential for analyzing the efficacy and safety of RDN and for exploring the reasons for the failure of this procedure in Simplicity HTN-3 study [15].

Our research aimed to assess acute changes in morphology and vascular relaxation function immediately after radiofrequency catheter-based RDN in a pig model.

## Methods

### Animals

The study was implemented using 6 8-month-old male Bama mini-pigs weighing 20 kg, which were provided by the Beijing Shi Chuang Century Minipig Breeding Base. The pigs housed individually in a special room with a suitable temperature (23 ± 1 °C) and humidity (50 ± 5%) had access to a high-fat diet containing 15% butter, 5% peanut oil and 80% basal feed, with a feeding guideline of 5% of its body weight every day. All animal experimental procedures were approved by the Institutional Laboratory Care and Use Committee of Zhengzhou University for Medical Research and were compliant with regulatory guidelines for the care of laboratory animals.

### Experimental design and preparation

The pigs were randomly divided into two groups, the RDN group (*n* = 3) and the Sham-RDN group (*n* = 3). We performed baseline renal arterial angiography for all the animals. In the RDN group, the conventional temperature-controlled cardiac radiofrequency catheter-based RDN was applied unilaterally. The same procedure was utilized in the Sham-RDN group except for the ablation. All pigs underwent angiography again immediately after the procedure and were then euthanized. Subsequently, the renal arteries were excised for the vascular tension, histology and molecular biology analyses.

### Renal arterial angiography and RDN perioperative period

Anesthesia was achieved with a combination of midazolam (0.5 mg/kg, En Hua Pharmaceuticals, Xuzhou, China) and atropine (0.025 mg/kg, Sui Cheng Pharmaceuticals, Zhengzhou, China) administered intramuscularly. An injectable etomidate emulsion (En Hua Pharmaceuticals, Xuzhou, China) and dexmedetomidine hydrochloride (Guo Rui Pharmaceuticals, Chengdu, China) were continuously pumped at a rate of 15 ml/h to maintain the anesthesia. The femoral arteries were punctured by the vascular incision method. The incision was located in the groin region and directed toward the midpoint of the last two nipple connections. A 7F sheath (Cordis Corporation, Florida, USA) was inserted into the artery and fixed. Then, each pig was administered heparin at 100 U/kg, which was repeated once per hour. The angiographic catheter (Cordis Corporation, Florida, USA) was introduced into the abdominal aortic region of the origin of the renal artery. We performed angiography using a standard contrast agent (Bayer Schering Pharma AG, Leverkusen, Germany) to assess the feasibility of the RDN program and obtain baseline imaging data. The angiographic catheter was withdrawn, followed by insertion of the temperature-controlled cardiac radiofrequency catheter (NS7TCDL174HS, Biosense Webster, California, USA), which was connected to a generator (Johnson & Johnson, New Jersey, USA). Subsequently, the RDN procedure was performed. Five radiofrequency ablation sites were selected from the distal to the proximal segments of the renal artery. The interval of the ablation points was 5 mm, and the RDN was performed in a spiral manner. The generator parameters used for the radiofrequency ablation were as follows: energy, 8 W and time at each site, 120 s [2]. After the procedure, the radiofrequency catheter was removed, and angiography was performed again to check the anatomical structure of the renal arteries. The same procedure except for the ablation was performed on animals in the Sham-RDN group. Finally, the pigs were euthanized, and the renal arteries were excised.

### Histopathological examination of the renal arteries and nerves in the ablated versus non-ablated pigs

The renal arteries were collected and evaluated by histological staining. Each renal artery was fixed for 24 h in 4% paraformaldehyde. After fixation, the tissues were washed, dehydrated by soaking in a concentration gradient (75, 85, 90, 95 and 100%) of alcohol, and finally cleared in xylene. Then, the tissues were embedded in paraffin wax and cut into 5-μm-thick sections at a 200-μm interval from the distal (kidney) to proximal (abdominal aorta) region [20]. The samples were then subjected to hematoxylin and eosin (HE), Verhoeff’s Van Gieson, and Masson staining.

### Histological evaluation

The renal arteries and adjacent nerve bundles were observed on the prepared slides, and 3 random fields of view were imaged for further analysis under a 20× objective by optical microscopy (Leica Biosystems, Wetzlar, Germany). The effect of the treatment on the nerves was divided into five levels for assessment by semi-quantitative scoring [21]. No abnormal findings indicated a grade of 0. Minimal damage, i.e., a grade of 1, was considered with the observation of nerves with minor damage, insignificant perineuronal inflammation or hemorrhage and limited endoneuronal damage (e.g., fibroplasia with little to no vacuolization, pyknosis or increase in cellularity). Mild damage, i.e., a grade of 2, was considered with the observation of more obvious or more relevant changes in nerve bundles, with increased cellularity, neurological inflammation, fibrosis or endogenous changes (e.g., vacuolization, pyknotic nuclei or digestive cavities). Moderate injury (i.e., grade 3) appeared as more pronounced and frequent changes than those of grade 2 in terms of perineuronal inflammation, fibrosis and endoneuronal damage, including swelling of endoneuronal tissue. Serious harm (i.e., grade 4) appeared as significant perineural inflammation or fibrosis, or even nerve structure loss, necrosis and axonal retraction [21]. The injury to the treated renal artery can be evaluated by changes in the intima, medial and adventitia revealed by HE staining. Masson and Verhoeff’s Van Gieson staining can be used to evaluate medial collagen fibers and elastic fibers, respectively. 3 microscopic fields (× 200) were randomly chosen in each minipig, and the collagen volume fraction and elastin volume fraction were analyzed using Image-Pro Plus 6.0 (Media Cybernetics, Inc., Rockville, MD, USA).

### Immunohistochemical processing

The complete and post-RDN renal artery paraffin sections were evaluated by immunohistochemistry. Immunohistochemical staining against von Willebrand factor (vWF) (1:300; ab6994, Abcam, Cambridge, UK) and α-smooth muscle actin (α-SMA) (1:2000; GB13044, Servicebio, Wuhan, China) were used to reflect injury to the endothelial cells and the media, respectively. Paraffin sections were immunostained for nerve tissue in the stromal elements using S-100 protein primary antibody (1:100; ab868, Abcam, Cambridge, UK). Immunostaining against tyrosine hydroxylase (TH) (1:200; ab75875, Abcam, Cambridge, UK) reflected the presence of functionally intact sympathetic axons within the nerve fascicles. HRP-labeled goat anti-rabbit and rat antibodies (G1211, Servicebio, Wuhan, China) and a DAB chromogenic kit (G1211, Servicebio, Wuhan, China) were used to perform the immunohistochemical assays [22].

### In vitro vascular relaxation function study

Vascular relaxation function was assayed as previously reported [23, 24]. The adipose and connective tissues of the renal arteries were stripped in Krebs solution at 4 °C with a pH of 7.4 containing the following (mmol/L): 118.3 NaCl, 4.7 KCl, 1.2 MgSO_4_, 1.2 KH_2_PO_4_, 2.5 CaCl_2_, 25 NaHCO_3_, 0.026 calcium disodium EDTA, and 5.5 glucose. Two 5-mm-long vascular rings from the renal artery were cut and were analyzed per pig. Renal arterial rings were mounted between two hooks connected to the tension transducer (JZJ01H, Chengdu Instrument Factory, Chengdu, China) and were placed in a water bath holding 6 ml of Krebs solution at 37 °C constantly maintaining 95% O_2_ and 5% CO_2_. The resting tension of the arterial rings was adjusted to 3 g, and the solution was replaced every 10 min. After 2 h of equilibration, the vascular rings were exposed to 60 mmol/L KCl solution to shrink. Then, the rings stood in a water bath for 30 min after elution and were treated. The arterial rings were pre-contracted with 6 × 10^− 6^ mol/L norepinephrine (NE) (Yuan Da Medicine, Wuhan, China) and then relaxed by a 10^− 9^–10^− 5^ mol/L concentration gradient of sodium nitroprusside (SNP) (Hong Yuan Pharmaceuticals, Dongguan, China). Tension was recorded by the multi-channel physiological signal acquisition and processing system (BM6420BD, Chengdu Instrument Factory, Chengdu, China). The relaxation rate was calculated by the relaxation percentage of the NE-induced pre-contraction. The relaxation-response curve was obtained by Prism 5.0 (GraphPad Software, California, USA) to contrast and analyze the relaxation rates of the renal arteries.

### Western blot analysis

For protein expression analysis, renal arteries were washed 2–3 times with cold PBS and homogenized by liquid nitrogen milling. The supernatant protein concentration was measured using a BCA Protein Assay Kit (G2026, Servicebio, Wuhan, China). The protein was separated on an 8–10% gel by SDS-PAGE and transferred to PDVF membranes at a constant current of 300 mA for half an hour. The membranes were then blocked for an hour by 5% skim milk dissolved in 0.5% TBST. Subsequently, the membranes were incubated at 4 °C overnight with primary rabbit polyclonal anti-endothelial nitric oxide synthase (eNOS; 1:1000; ab5589, Abcam, Cambridge, UK), rabbit polyclonal anti-phosphorylated eNOS Ser^1177^ (1:1000;9571, CST, Boston, USA), rabbit polyclonal anti-acetophenone (AKT;1:1000; 9272, CST, Boston, USA), rabbit polyclonal anti-phosphorylated AKT Ser^473^ (1:1000; 9271, CST, Boston, USA) and mouse monoclonal anti-glyceraldehyde phosphate dehydrogenase (GAPDH;1:25000; GB13002-m-1, Servicebio, Wuhan, China) antibodies. The membranes were washed three times with TBST buffer at room temperature and then incubated with peroxidase-labeled goat anti-rabbit IgG (H + L) secondary antibody (1:3000; 074–1506, KPL, Maryland, USA) and peroxidase-labeled goat anti-mouse IgG (H + L) secondary antibody (1:3000; 074–1806, KPL, Maryland, USA) for half an hour [24]. AlphaEaseFC software (Alpha Innotech, California, USA) was used to analyze the optical density of the target bands.

### Statistical analysis

The experimental data were analyzed using the SPSS 20.0 software package. Data following a normal distribution were analyzed by independent sample t-tests and two-way repeated ANOVA, and the Mann-Whitney U-rank test was used to analyze data that did not follow a normal distribution. *P* values less than 0.05 were considered significant. Data are presented as the mean and standard deviation.

## Results

### Comparison of angiograms of pre- and post-RDN renal arteries

**Fig. 1:**
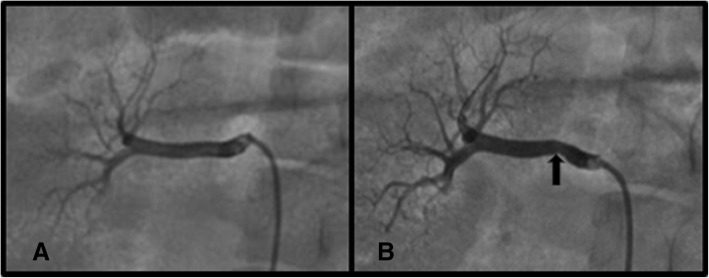
Angiography and RDN perioperative period. (**a**) pre-RDN, (**b**) post-RDN. Black arrow indicates the vasospasm site. Abbreviations: RDN, renal denervation

Compared with the pre-RDN arteries (Fig. 1a), segments of the post-RDN arteries exhibited vasospasm by angiography in all 3 pigs of RDN group (Fig. 1b); however, we did not find other changes, such as dissection or thrombus or aneurysm formation, in the acute phase after RDN. The vasospasm was of a mild or moderate degree, which would not affect renal perfusion over a short period.

### Acute changes in renal arterial wall assessed immediately after RDN

**Fig. 2:**
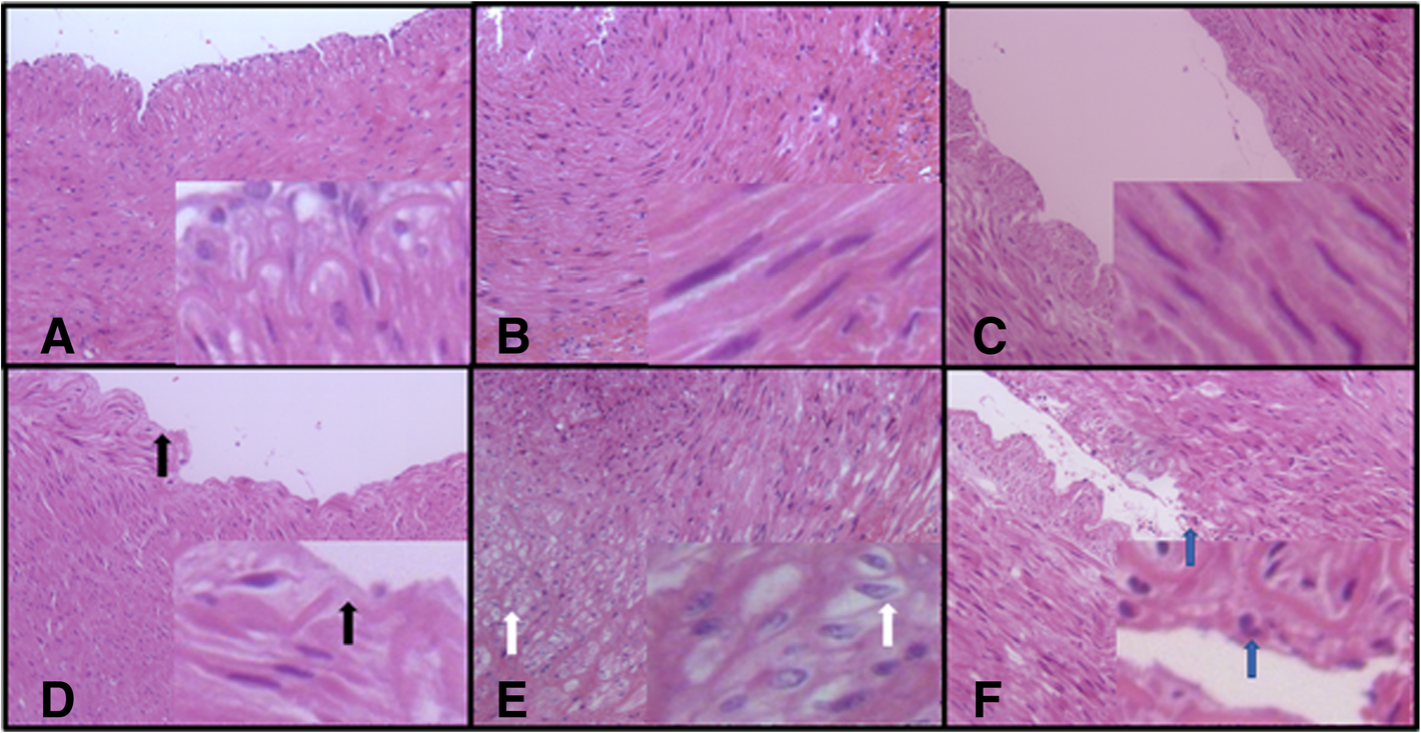
Representative HE staining images of renal arterial wall (× 200). (**a**-**c**) The Sham-RDN group, (**d**-**f**) the RDN group. Black arrows indicate internal elastic lamina rupture, white arrows indicate vacuolar degeneration, and blue arrows indicate neutrophil infiltration in lesion area. Abbreviations: RDN, renal denervation; HE, hematoxylin and eosin

**Fig. 3:**
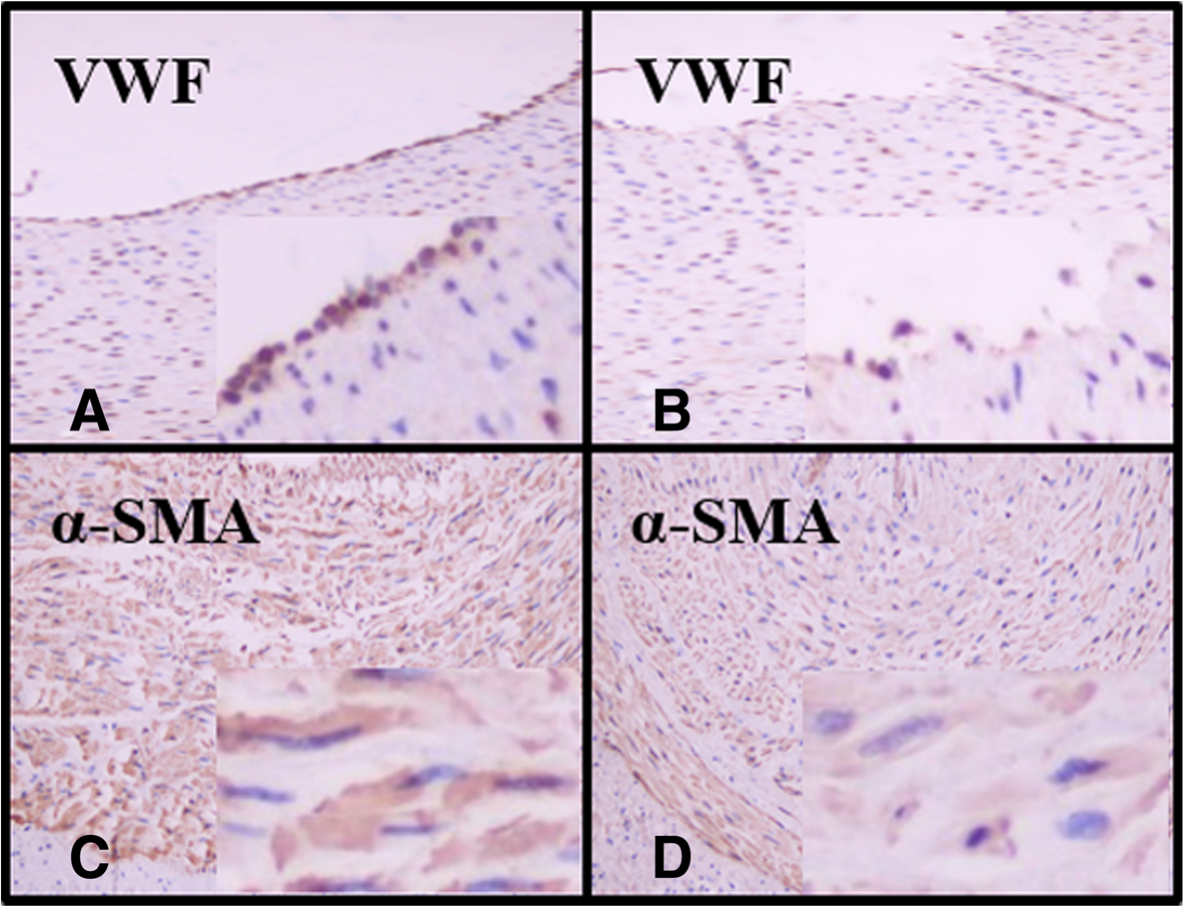
Staining of endothelial cells and smooth muscle cells (× 200). (**a** and **c**) Untreated renal artery, (**b** and **d**) treated renal artery. Abbreviations: vWF, von Willebrand factor; α-SMA, α-smooth muscle actin

The HE staining results revealed internal elastic lamina rupture (Fig. 2d), vacuolar degeneration of smooth muscle cells (Fig. 2e) and neutrophil infiltration (Fig. 2f) post-RDN; these changes were not observed in the corresponding areas of the Sham-RDN group (Fig. 2a, b, c). While the Sham-RND group showed strongly positive staining (Fig. 3a, c), vWF staining in the RDN group revealed damaged or even absent endothelial cells (Fig. 3b), and the α-SMA staining intensity in the RDN group was weakly positive within the lesion area (Fig. 3d).

### Acute changes in nerves fibers assessed immediately after RDN

**Fig. 4:**
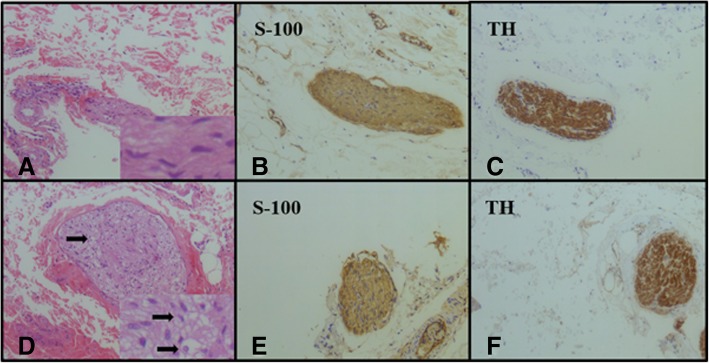
Representative histological images of nerves (× 200). (**a**-**c**) The Sham-RDN group, (**d**-**f**) the RDN group. Black arrows indicate neuro-vacuolar degeneration sites. Abbreviations: RDN, renal denervation; TH, tyrosine hydroxylase

**Fig. 5:**
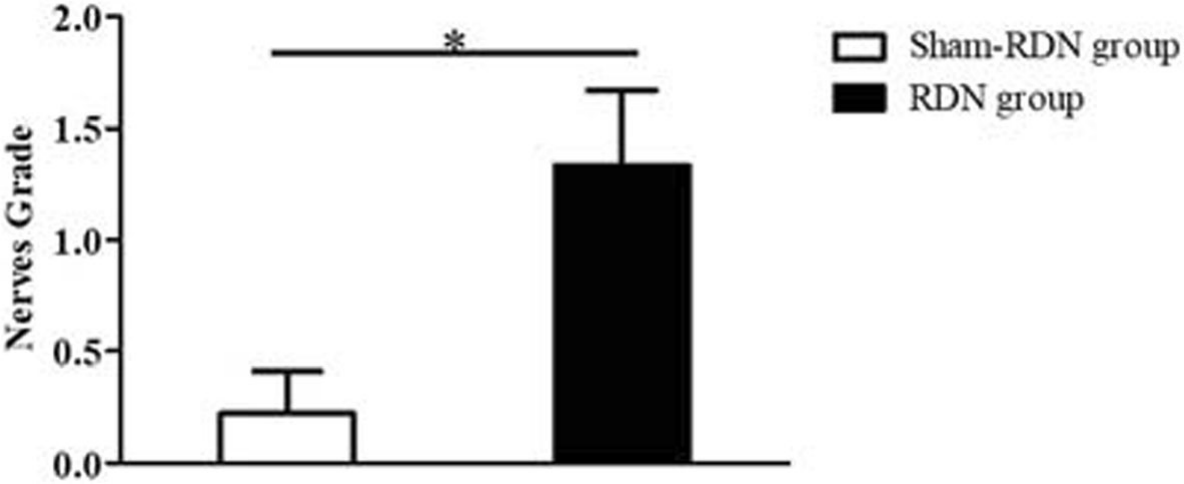
Comparison of nerve injury grade in the Sham-RDN and RDN groups. Data are expressed as the mean ± standard deviation. *P < 0.05, *n* = 3 per group. Abbreviations: RDN, renal denervation

**Fig. 6:**
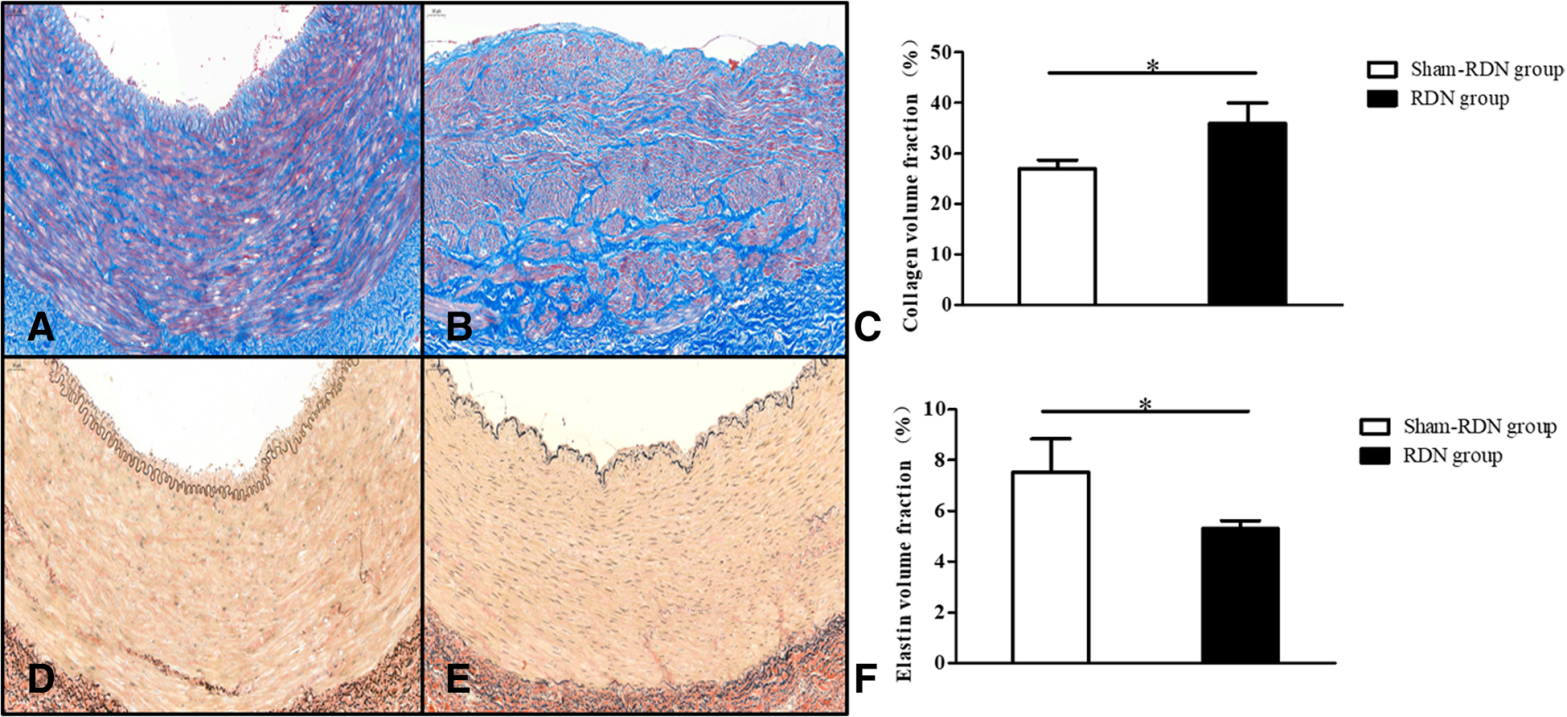
Microphotographs of renal arterial fiber sections (× 200). Masson staining showing collagen fibers (blue) of arterial segments (**a**) without RDN and (**b**) with RDN. Verhoeff’s Van Gieson staining showing elastic fibers (black) of (**d**) untreated and (**e**) treated arterial segments. Comparison of collagen volume fraction (**c**) and elastin volume fraction (F) between the two groups. Abbreviations: RDN, renal denervation

Compared with the Sham-RDN group (Fig. 4a), the RDN group showed increased axonal vacuolated denaturation, as demonstrated by HE staining (Fig. 4d). The S-100 antibody staining (Fig. 4b, e) and TH antibody staining (Fig. 4c, f) results were positive in both groups. Regarding the semi-quantitative scoring of nerve injury (Fig. 5), the grades of nerve injury in the Sham-RDN and RDN groups were 0.22 ± 0.19 and 1.33 ± 0.34 (*P* = 0.046), respectively. The nerves in the Sham-RDN group showed no abnormal findings or minimal damage; however, the nerves in the RDN group showed mild damage. As observed in the pigs treated RDN immediately after sacrifice, the collagen fibers significantly exhibited mild hyperplasia compared to Sham-RDN group (Fig. 6a-c,35.84 ± 4.14% vs. 26.95 ± 1.76%, *p* = 0.027). The elastic fibers were significantly irregular, separated and broken in RDN group compared to Sham-RDN group (Fig. 6d-f, 5.30 ± 0.31% vs. 7.52 ± 1.31%, *p* = 0.047).

### Comparison of renal arterial vascular tension in vitro with and without RDN

**Fig. 7:**
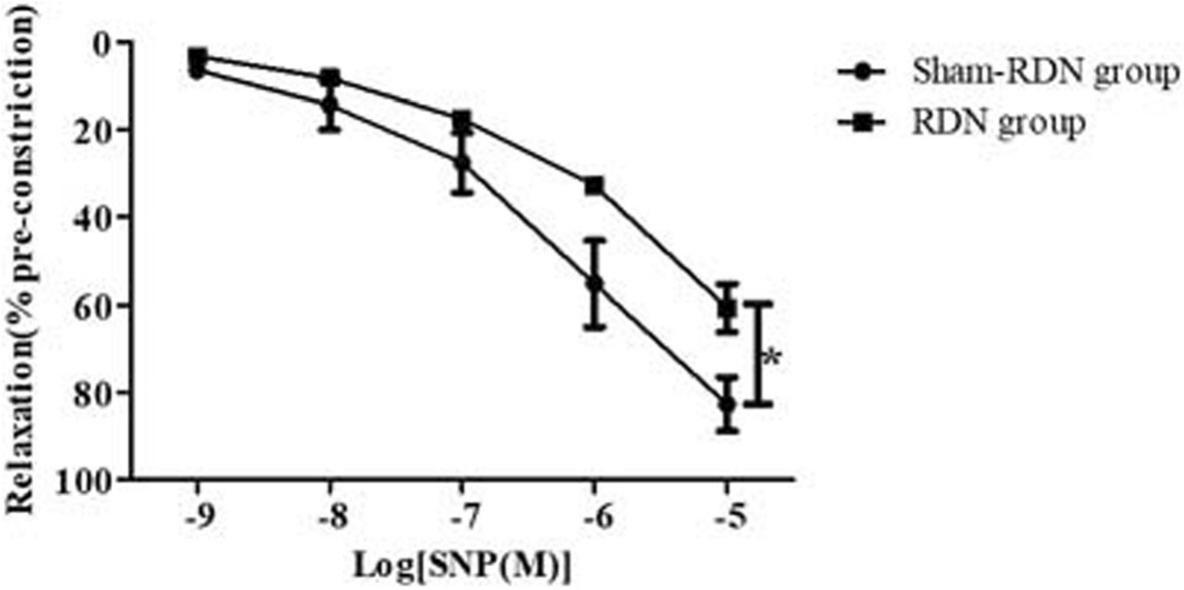
Endothelium-independent vascular relaxation response to SNP in the two groups. Data are expressed as the mean ± standard deviation. *P < 0.05, *n* = 3 per group. Abbreviations: RDN, renal denervation; SNP, sodium nitroprusside

The in vitro vascular tension measurements showed that endothelium-independent vascular relaxation function was significantly decreased (*P* = 0.023) in the SNP concentration gradient in the RDN group (Fig. 7, Additional file 1) compared to Sham-RDN group, which was coincident with smooth muscle cell degeneration and weakly positive α-SMA staining, as mentioned above (Figs. 2e and 3d).

### Acute effect of RDN on endothelial function signaling at the molecular level

**Fig. 8:**
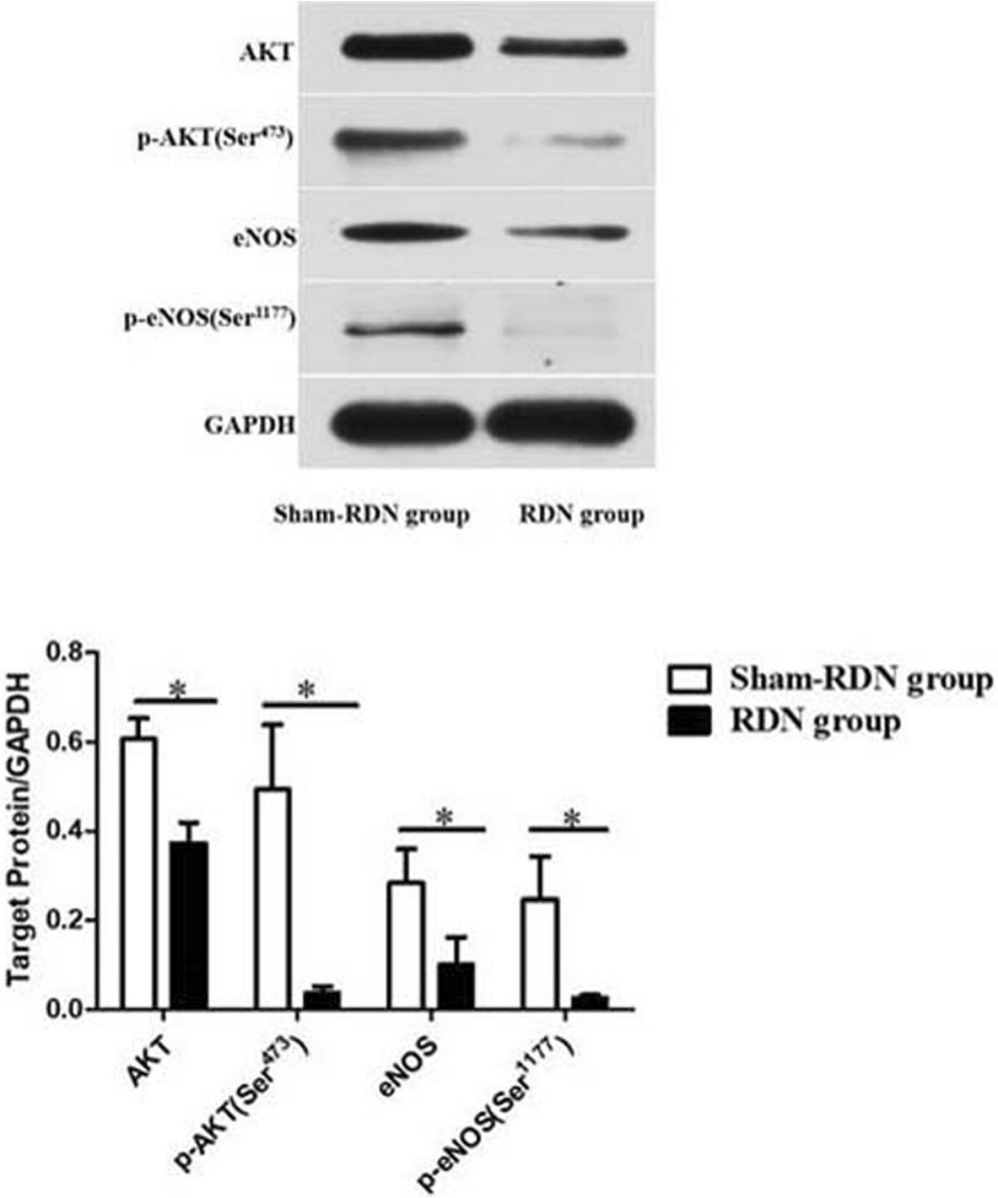
AKT, eNOS, phosphorylation of eNOS Ser^1177^ and AKT Ser^473^ expression with and without RDN. The results are expressed as the mean ± standard deviation. *P < 0.05, *n* = 3 per group. Abbreviations: AKT, acetophenone; eNOS, endothelial nitric oxide synthase; RDN, renal denervation; GAPDH, glyceraldehyde phosphate dehydrogenase

The effects of RDN on endothelium-independent vascular relaxation were significant (*P* = 0.023, Fig. 7), and this might affect the in vitro tension measurements of the endothelium-dependent vascular relaxation function. Therefore, we only examined the acute effects of RDN on the endothelial nitric oxide (NO) synthesis pathway. Compared with the Sham-RDN group, the protein expression of AKT, p- AKT Ser^473^, eNOS, and p-eNOS Ser^1177^ was significantly decreased in the RDN group (*P* = 0.046, *P* = 0.049, *P* = 0.032, P = 0.046, respectively), and the endothelial NO synthesis pathway was obviously inhibited (Fig. 8).

## Discussion

In this study, we clearly showed the presence of acute changes in morphology and vascular relaxation function immediately after temperature-controlled radiofrequency catheter-based RDN in a porcine model. The radiofrequency catheter released high energy which penetrated the intima and media of the renal arteries and destroyed functional sympathetic nerve fibers in the adventitia. In the acute phase, RDN caused mild and moderate vasospasm, but no arterial dissection or stenosis was observed by angiography, and these findings are consistent with those of a previous report [17]. After RDN, we observed that nerve bundles were mildly damaged and showed endogenous changes, such as vacuolization and pyknotic nuclei but without karyorrhexis, as well as the formation of digestive cavities mentioned by Kenichi Sakakura et al. [21]. These differences may result from the individual anatomical differences. Sakakura et al. [25] illustrated that the peri-arterial renal nerve size and distance from the renal artery lumen were indefinite, which affected the efficiency of the RDN procedure. In this study, we immunostained against S-100 protein (a marker of Schwann and sustentacular cells) and TH (converts tyrosine to DOPA and is a marker of norepinephrine synthesis). Compared with the Sham-RDN group, there were no significant changes in the immunostaining intensity of the neuronal markers S-100 and TH in the RDN group. Therefore, immunostaining in the acute phase cannot better reflect the success of RDN, which may be due to the certain stage of axonal degeneration occurring in the necrotic nerves [21]. Because of our immediate sampling to evaluate the acute morphology alternations of renal arteries after RDN, the short interval after the operation and the use of anesthesia may lead to norepinephrine determination inaccuracies; thus, we used a semi-quantitative scoring system to immediately assess the efficacy of the procedure. The results showed a significant difference between 2 groups (*P* < 0.05), which facilitated the preclinical study of the renal arterial histological progression after RDN.

Another focus of this study was the post-RDN effects on the endothelium and vascular relaxation function. Immunohistochemical staining against vWF revealed severely reduced or even absent post-RDN levels. Steigerwald et al. [17] also reported this result as well as reendothelialization in the subacute phase after approximately 10 days, as demonstrated by the nearly complete vWF staining within the lesion. In addition, Steigerwald et al. [17] mentioned that the intact internal elastic lamina and the remaining collagen fibers were able to maintain vessel wall integrity. However, the recovery of the endothelial function and vascular relaxation function should be considered. Thus far, the safety endpoints of RDN using the different protocols involved in the study [26] were mainly renal arterial radiologically morphological lesions, renal dysfunction and adverse outcomes such as renal vascular dissection, aneurysm and thrombosis, and there have been no reports on the study of endothelial and smooth muscle cell function after RDN.

NO is synthesized from L-arginine by eNOS and initiates vascular relaxation by activating soluble guanylate cyclase in vascular smooth muscle cells [27]. Our findings demonstrate that RDN caused injury to the vascular intima and media; and the in vitro endothelium-independent tension assay showed that renal arterial relaxation function decreased after the RDN. This result is due to elastic fiber breakage, vascular smooth muscle cell degeneration and necrosis, which was also consistent with the thin media and reduced myofilament density in ablated medial smooth muscle cells after RDN in Sakaoka’s study [28]. Unfortunately, we did not detect endothelium-dependent vascular relaxation function and only detected AKT/eNOS pathway-related protein expression, which explained the endothelial dysfunction molecularly. Because the effects of smooth muscle cell damage can disturb the detection of endothelium-dependent vascular relaxation function. Of course, to observe the imaging and histological changes immediately after catheter-based renal sympathetic denervation, we did not wait for or interfere with vasospasm recovery, which prevented us from measuring vascular relaxation in vivo and over the long term. Downregulation of the NO synthesis pathway after endothelial injury is likely to be associated with vasospasm at the site of ablation [29]. Schmid et al. [30] detected no vascular or parenchymal complications after 6 and 12 months of RDN, but a case report showed secondary rise in blood pressure and a haemodynamically relevant renal arterial stenosis after 6 months of RDN [31]; therefore, endothelial dysfunction may have an adverse impact on renal arteries over a longer period.

A recent study [20] supported potential applications for saline-irrigated catheter (SIC) ablation and, at histopathological levels, found that SIC caused less intimal hyperplasia, deeper penetration and more medial hyperplasia than temperature-controlled catheter ablation for RDN. However, changes in vascular and endothelial function produced by more deeply penetrating lesions using SIC remain to be explored. Destruction of the vascular wall integrity can gradually increase the concentration of vascular superoxide [32], which also has a negative effect on the response to endogenous and exogenous vascular relaxation [33]. Moorhouse et al. [34] suggested that endothelial dysfunction presenting a reduced release of endothelium-derived relaxation factors such as NO, and the increased release of endothelial-derived contractility factors such as endothelin-1, caused abnormal vasoconstriction, elevated blood pressure which increased the risk of hypertension recurrence and an unsuccessful RDN procedure, and also was an early sign of the development of atherosclerosis [35].

## Conclusions

Our study showed histological changes in renal arteries and nerves in the acute phase after RDN. It was feasible to immediately evaluate the success of the RDN procedure using a semi-quantitative method for scoring nerve injury. However, from a clinical perspective, we needed to find effective and completely ablated markers to immediately determine the success of RDN. In addition, this study was the first to propose changes in vascular relaxation function after RDN. we found that the RDN procedure caused the destruction of the renal artery integrity, leading to acute-phase endothelial and vascular relaxation dysfunction. Therefore, it is important to assess the safety of the RDN procedure in the short and long term from multiple perspectives and to take steps, such as monitoring endothelial function and anti-endothelial dysfunction drugs, to prevent and treat complications in time.

## Limitations

There were several limitations in our study. First, in this study, we used healthy and normotensive pigs, which cannot completely reflect the sympathetic hyperactivity as well as vascular and even endothelial function of people with hypertension. However, persistently elevated blood pressure in patients with resistant hypertension can cause the premature aging of endothelial cells and vasodilation dysfunction [36], which may further increase the risk of adverse events after RDN. In addition, pig models are widely used in preclinical studies because porcine anatomy is similar to that of humans. Second, this study provides a descriptive analysis of the experimental results, although with a small sample size, there was still a limited statistical significance. Third, we only observed changes in endothelial and vascular relaxation function in pigs that were sacrificed immediately after RDN and did not study late-stage progression. However, it must also be considered that endothelial and vascular relaxation dysfunction may affect the prognosis of hypertensive patients. 

## Additional file

Additional file 1: Comparison of mean endothelium-independent vasodilation rate changes at different concentrations of all pigs was as follows. (DOC 15 kb)

## Abbreviations

AKT: Acetophenone
eNOS: Endothelial nitric oxide synthase
GAPDH: Glyceraldehyde phosphate dehydrogenase
HE: Hematoxylin and eosin
NO: Nitric oxide
RDN: Renal denervation
SNP: Sodium nitroprusside
TH: Tyrosine hydroxylase
vWF: von Willebrand factor
α-SMA: α-Smooth muscle actin

## References

[1] Dzau VJ, Antman EM, Black HR, Hayes DL, Manson JE, Plutzky J, Popma JJ, Stevenson W (2006). The cardiovascular disease continuum validated: clinical evidence of improved patient outcomes: part i: pathophysiology and clinical trial evidence (risk factors through stable coronary artery disease). Circulation.

[2] Krum H, Schlaich M, Whitbourn R, Sobotka PA, Sadowski J, Bartus K, Kapelak B, Walton A, Sievert H, Thambar S, Abraham WT, Esler M (2009). Catheter-based renal sympathetic denervation for resistant hypertension: a multicentre safety and proof-of-principle cohort study. Lancet.

[3] Symplicity HTNI (2011). Catheter-based renal sympathetic denervation for resistant hypertension: durability of blood pressure reduction out to 24 months. Hypertension.

[4] James PA, Oparil S, Carter BL, Cushman WC, Dennison-Himmelfarb C, Handler J, Lackland DT, LeFevre ML, MacKenzie TD, Ogedegbe O, Smith SC, Svetkey LP, Taler SJ, Townsend RR, Wright JT, Narva AS, Ortiz E (2014). 2014 evidence-based guideline for the management of high blood pressure in adults: report from the panel members appointed to the eighth joint national committee (jnc 8). Jama.

[5] Persell SD (2011). Prevalence of resistant hypertension in the United States, 2003-2008. Hypertension.

[6] Vink EE, Blankestijn PJ (2012). Evidence and consequences of the central role of the kidneys in the pathophysiology of sympathetic hyperactivity. Front Physiol.

[7] DiBona GF (2000). Nervous kidney. Interaction between renal sympathetic nerves and the renin-angiotensin system in the control of renal function. Hypertension.

[8] Sobotka PA, Mahfoud F, Schlaich MP, Hoppe UC, Bohm M, Krum H (2011). Sympatho-renal axis in chronic disease. Clinical research in cardiology : official journal of the German Cardiac Society.

[9] Symplicity HTNI, Esler MD, Krum H, Sobotka PA, Schlaich MP, Schmieder RE, Bohm M (2010). Renal sympathetic denervation in patients with treatment-resistant hypertension (the symplicity htn-2 trial): a randomised controlled trial. Lancet.

[10] Prochnau D, Lucas N, Kuehnert H, Figulla HR, Surber R: Catheter-based renal denervation for drug-resistant hypertension by using a standard electrophysiology catheter. EuroIntervention : journal of EuroPCR in collaboration with the working group on interventional cardiology of the European Society of Cardiology 2012;7:1077–1080. 10.4244/EIJV7I9A171 21959556

[11] Worthley SG, Tsioufis CP, Worthley MI, Sinhal A, Chew DP, Meredith IT, Malaiapan Y, Papademetriou V (2013). Safety and efficacy of a multi-electrode renal sympathetic denervation system in resistant hypertension: the enlightn i trial. Eur Heart J.

[12] Esler MD, Bohm M, Sievert H, Rump CL, Schmieder RE, Krum H, Mahfoud F, Schlaich MP (2014). Catheter-based renal denervation for treatment of patients with treatment-resistant hypertension: 36 month results from the symplicity htn-2 randomized clinical trial. Eur Heart J.

[13] Krum H, Schlaich MP, Sobotka PA, Bohm M, Mahfoud F, Rocha-Singh K, Katholi R, Esler MD (2014). Percutaneous renal denervation in patients with treatment-resistant hypertension: final 3-year report of the symplicity htn-1 study. Lancet.

[14] Mahfoud F, Luscher TF, Andersson B, Baumgartner I, Cifkova R, Dimario C, Doevendans P, Fagard R, Fajadet J, Komajda M, Lefevre T, Lotan C, Sievert H, Volpe M, Widimsky P, Wijns W, Williams B, Windecker S, Witkowski A, Zeller T, Bohm M (2013). European Society of C: expert consensus document from the european society of cardiology on catheter-based renal denervation. Eur Heart J.

[15] Bhatt DL, Kandzari DE, O'Neill WW, D'Agostino R, Flack JM, Katzen BT, Leon MB, Liu M, Mauri L, Negoita M, Cohen SA, Oparil S, Rocha-Singh K, Townsend RR, Bakris GL, Investigators SH-: A controlled trial of renal denervation for resistant hypertension. N Engl J Med 2014;370:1393–1401. 10.1056/NEJMoa1402670 24678939

[16] Rippy MK, Zarins D, Barman NC, Wu A, Duncan KL, Zarins CK (2011). Catheter-based renal sympathetic denervation: chronic preclinical evidence for renal artery safety. Clinical research in cardiology : official journal of the German Cardiac Society.

[17] Steigerwald K, Titova A, Malle C, Kennerknecht E, Jilek C, Hausleiter J, Nahrig JM, Laugwitz KL, Joner M (2012). Morphological assessment of renal arteries after radiofrequency catheter-based sympathetic denervation in a porcine model. J Hypertens.

[18] Sakakura K, Tunev S, Yahagi K, O'Brien AJ, Ladich E, Kolodgie FD, Melder RJ, Joner M, Virmani R (2015). Comparison of histopathologic analysis following renal sympathetic denervation over multiple time points. Circulation Cardiovascular interventions.

[19] Taborsky M, Richter D, Tonar Z, Kubikova T, Herman A, Peregrin J, Cervenkova L, Huskova Z, Kopkan L (2017). Early morphologic alterations in renal artery wall and renal nerves in response to catheter-based renal denervation procedure in sheep: difference between single-point and multiple-point ablation catheters. Physiol Res.

[20] Wang Z, Chen S, Zhou T, Su L, Ling Z, Fan J, Chen W, Du H, Lu J, Xu Y, Tan Z, Yang H, Hu X, Li C, Yan X, Hu G, Liu C, Yin Y (2015). Comparison of saline-irrigated catheter vs. temperature-controlled catheter for renal denervation in a canine model. Am J Hypertens.

[21] Sakakura K, Ladich E, Edelman ER, Markham P, Stanley JR, Keating J, Kolodgie FD, Virmani R, Joner M (2014). Methodological standardization for the pre-clinical evaluation of renal sympathetic denervation. JACC Cardiovascular interventions.

[22] Feng Q, Lu C, Wang L, Song L, Li C, Uppada RC (2017). Effects of renal denervation on cardiac oxidative stress and local activity of the sympathetic nervous system and renin-angiotensin system in acute myocardial infracted dogs. BMC Cardiovasc Disord.

[23] Sun Y, Li J, Xiao N, Wang M, Kou J, Qi L, Huang F, Liu B, Liu K (2014). Pharmacological activation of ampk ameliorates perivascular adipose/endothelial dysfunction in a manner interdependent on ampk and sirt1. Pharmacol Res.

[24] Vilahur G, Casani L, Mendieta G, Lamuela-Raventos RM, Estruch R, Badimon L (2014). Beer elicits vasculoprotective effects through akt/enos activation. Eur J Clin Investig.

[25] Sakakura K, Ladich E, Cheng Q, Otsuka F, Yahagi K, Fowler DR, Kolodgie FD, Virmani R, Joner M (2014). Anatomic assessment of sympathetic peri-arterial renal nerves in man. J Am Coll Cardiol.

[26] Weber MA, Kirtane A, Mauri L, Townsend RR, Kandzari DE, Leon MB (2015). Renal denervation for the treatment of hypertension: making a new start, getting it right. Clin Cardiol.

[27] Vanhoutte PM, Shimokawa H, Feletou M, Tang EH (2017). Endothelial dysfunction and vascular disease - a 30th anniversary update. Acta Physiol.

[28] Sakaoka A, Takami A, Onimura Y, Hagiwara H, Terao H, Kumagai F, Matsumura K (2017). Acute changes in histopathology and intravascular imaging after catheter-based renal denervation in a porcine model. Catheterization and cardiovascular interventions : official journal of the Society for Cardiac Angiography & Interventions.

[29] Templin C, Jaguszewski M, Ghadri JR, Sudano I, Gaehwiler R, Hellermann JP, Schoenenberger-Berzins R, Landmesser U, Erne P, Noll G, Luscher TF (2013). Vascular lesions induced by renal nerve ablation as assessed by optical coherence tomography: pre- and post-procedural comparison with the simplicity catheter system and the enlightn multi-electrode renal denervation catheter. Eur Heart J.

[30] Schmid A, Schmieder R, Lell M, Janka R, Veelken R, Schmieder RE, Uder M, Ott C (2016). Mid-term vascular safety of renal denervation assessed by follow-up mr imaging. Cardiovasc Intervent Radiol.

[31] Vonend O, Antoch G, Rump LC, Blondin D (2012). Secondary rise in blood pressure after renal denervation. Lancet.

[32] Azevedo LC, Pedro MA, Souza LC, de Souza HP, Janiszewski M, da Luz PL, Laurindo FR (2000). Oxidative stress as a signaling mechanism of the vascular response to injury: the redox hypothesis of restenosis. Cardiovasc Res.

[33] Heitzer T, Wenzel U, Hink U, Krollner D, Skatchkov M, Stahl RA, MacHarzina R, Brasen JH, Meinertz T, Munzel T (1999). Increased nad(p) h oxidase-mediated superoxide production in renovascular hypertension: evidence for an involvement of protein kinase c. Kidney Int.

[34] Moorhouse RC, Webb DJ, Kluth DC, Dhaun N (2013). Endothelin antagonism and its role in the treatment of hypertension. Curr Hypertens Rep.

[35] Davignon J, Ganz P (2004). Role of endothelial dysfunction in atherosclerosis. Circulation.

[36] Konukoglu D, Uzun H (2017). Endothelial dysfunction and hypertension. Adv Exp Med Biol.

