# Supplementary material for: Acute changes in morphology and renal vascular relaxation function after renal denervation using temperature-controlled radiofrequency catheter
Source: BMC Cardiovasc Disord. 2019 Mar 22;19:67. doi: 10.1186/s12872-019-1053-z (PMC6431051; doi:10.1186/s12872-019-1053-z)
Supplement: Supplementary file 1 — Comparison of mean endothelium-independent vasodilation rate changes at different concentrations of all pigs was as follows. (DOC 15 kb) [file 12872_2019_1053_MOESM1_ESM.doc]

Comparison of mean endothelium-independent vasodilation rate changes at different concentrations of all pigs was as follows:

|  | Sham-RDN group | | | RDN group | | |
| --- | --- | --- | --- | --- | --- | --- |
| SNP  (mmol/L) | Pig1 | Pig2 | Pig3 | Pig4 | Pig5 | Pig6 |
| **10^^-9^** | 8.3% | 5.0% | 6.3% | 2.4% | 4.8% | 2.9% |
| **10^^-8^** | 20.8% | 10.0% | 12.5% | 7.1% | 9.5% | 8.6% |
| **10^^-7^** | 35.4% | 22.5% | 25.0% | 19.0% | 16.7% | 17.1% |
| **10^^-6^** | 64.6% | 45.0% | 56.3% | 31.0% | 33.3% | 34.3% |
| **10^^-5^** | 89.6% | 77.5% | 81.3% | 54.8% | 61.9% | 65.7% |

Abbreviations: RDN, renal denervation; SNP, sodium nitroprusside.
